# *Lutzomyia umbratilis*, the Main Vector of *Leishmania guyanensis*, Represents a Novel Species Complex?

**DOI:** 10.1371/journal.pone.0037341

**Published:** 2012-05-18

**Authors:** Vera Margarete Scarpassa, Ronildo Baiatone Alencar

**Affiliations:** 1 Coordenação de Biodiversidade, Instituto Nacional de Pesquisas da Amazônia, Manaus, Amazonas, Brazil; 2 Programa de Pós-Graduação em Entomologia, Instituto Nacional de Pesquisas da Amazônia, Manaus, Amazonas, Brazil; Centro de Pesquisas René Rachou, Brazil

## Abstract

**Background:**

*Lutzomyia umbratilis* is an important *Leishmania guyanensis* vector in South America. Previous studies have suggested differences in the vector competence between *L. umbratilis* populations situated on opposite banks of the Amazonas and Negro Rivers in the central Amazonian Brazil region, likely indicating a species complex. However, few studies have been performed on these populations and the taxonomic status of *L. umbratilis* remains unclear.

**Methodology/Principal Findings:**

Phylogeographic structure was estimated for six *L. umbratilis* samples from the central Amazonian region in Brazil by analyzing mtDNA using 1181 bp of the *COI* gene to assess whether the populations on opposite banks of these rivers consist of incipient or distinct species. The genetic diversity was fairly high and the results revealed two distinct clades ( = lineages) with 1% sequence divergence. Clade I consisted of four samples from the left bank of the Amazonas and Negro Rivers, whereas clade II comprised two samples from the right bank of Negro River. No haplotypes were shared between samples of two clades. Samples within clades exhibited low to moderate genetic differentiation (*F*_ST_ = −0.0390–0.1841), whereas samples between clades exhibited very high differentiation (*F*_ST_ = 0.7100–0.8497) and fixed differences. These lineages have diverged approximately 0.22 Mya in the middle Pleistocene. Demographic expansion was detected for the lineages I and II approximately 30,448 and 15,859 years ago, respectively, in the late Pleistocene.

**Conclusions/Significance:**

The two genetic lineages may represent an advanced speciation stage suggestive of incipient or distinct species within *L. umbratilis*. These findings suggest that the Amazonas and Negro Rivers may be acting as effective barriers, thus preventing gene flow between populations on opposite sides. Such findings have important implications for epidemiological studies, especially those related to vector competence and anthropophily, and for vector control strategies. In addition, *L. umbratilis* represents an interesting example in speciation studies.

## Introduction

In the last three decades, molecular genetic markers have been widely used for elucidating the population structure and evolutionary genetics in insect vectors, and these studies have contributed to differentiate members of cryptic species complexes, primarily in malaria [1], [2], [3], leishmaniasis [4], [5], and Chagas [6], [7], [8] vectors. Cryptic species are discrete species that are difficult or sometimes impossible to distinguish morphologically [9], although their genetics, behavioral/ecological aspects, susceptibility to infection, and feeding habits (ranging from anthropophily to zoophily) often vary, defining their vector or non-vector status. Furthermore, species that diverged very recently complicates the understanding of reproductive isolation, evolutionary relationships and geographic distributions because gene flow may occur between them. In case of vectors, the potential gene flow between species or between different evolutionary units may have relevant epidemiological consequences, as it may facilitate inter-specific transfer of epidemiologically important genes or alleles and changes the transmission patterns of the diseases [10].

The main vector of *Leishmania guyanensis* Floch, 1954 that causes American Cutaneous Leishmaniasis (ACL), *Lutzomyia umbratilis* Ward & Fraiha, 1977 (Diptera: Psychodidae), occurs in northern South America, including Bolivia, Brazil, Colombia, French Guyana, Peru, Suriname and Venezuela [11], [12]. In Brazil, *L. umbratilis* has been registered in the Amazonian region [11] and in the state of Pernambuco [13] from the northeastern region. Consequently, populations of this species occupy extensive areas separated by geographic barriers, including the largest rivers, the Amazonas and Negro, in Amazonian Brazil. Additionally, sandflies have very limited dispersal capabilities, usually no more than 1 km [14], [15], are abundant in peridomestic environments of rural communities and therefore are distributed in discontinuous areas [16]. Taken together, these characteristics suggest that *L. umbratilis* populations are more susceptible to evolving into differentiated populations, incipient species and, ultimately, reproductively isolated species.

*Lutzomyia umbratilis* has been implicated in *Le. guyanensis* transmission in Brazil [17], [18], [19], French Guyana [20], [21], [22] and Venezuela [23]. In Amazonian Brazil, this species was incriminated in *Le. guyanensis* transmission in the states of Pará [17], [18], [19], Amazonas [24], [25], [26], [27], [28], Acre [29] and Rondônia [30]. In the municipality of Manaus (Amazonas), where *L. umbratilis* is recognized as the most important vector, the number of human cases of ACL was considered the highest in Brazil, comprising ∼57.4% of the autochthonous cases in the region [31]. In Peixoto de Azevedo, Mato Grosso, Brazil, an infection with *Leishmania braziliensis* Vianna, 1911 was identified in *L. umbratilis* [12]. Moreover, the susceptibility of this vector to *Leishmania* seems to vary in the central Amazonian Brazil region [25]. *Lutzomyia umbratilis* populations naturally infected with *Le. guyanensis* have been observed east of the Negro River and north of the Amazonas River; however, at south of the Amazonas River fluvial system have not been reported evidences of natural infections by *Leishmania* for this species [25]. Arias and Freitas [25] suggested that the fluvial system formed by the Amazonas, Solimões and Negro Rivers may act as a barrier to the *Le. guyanensis* transmission cycle, where *L. umbratilis* populations display distinct degrees of vector competence between the opposite sides. However, only a few studies have tested the role of this barrier in *L. umbratilis* genetic subdivision.

A biological analysis, under laboratory conditions, was conducted with two *L. umbratilis* populations from Manaus and Manacapuru (left and right sides of the Negro River, respectively) from Amazonas, Brazil. The results of this study revealed significant differences in the life cycle, fecundity, fertility, emergence degree and adult longevity between populations, which are indicative of intrinsic biological differences [32]. Subsequently, a study that combined morphology, chromosome and isozymes analysis of four *L. umbratilis* populations of this fluvial system showed significant differences in the bristle lengths of 4^th^ instar larvae and in the number and size of the spines of armature of the female genital atrium [33]. This latter has been a useful marker for distinguishing closely related species in sandflies [34]. Although polytene chromosome analysis was not possible, the metaphasic karyotype was 2n = 6. Isozymes did not reveal differences among populations. Isozymes evolve relatively slow rate likely due to negative selection and the amino acid codon wobble effect. Consequently, they are not informative markers for detecting incipient or recently diverged species [35]. Therefore, the taxonomic status of *L. umbratilis* remains unclear.

Genes encoding mitochondrial DNA (mtDNA) have been widely employed in population studies, molecular taxonomy and phylogenetic relationships of many organisms [36]. The rapid evolution and usually maternal inheritance of this marker makes it an attractive tool to evolutionary studies of recently diverged groups, where only the most rapidly evolving nucleotide sites accumulate substitutions. The mitochondrial genome of the majority of the animal taxa harbors 2 ribosomal RNA genes, 22 transfer RNA genes, and 13 protein-coding genes. The cytochrome oxidase subunit I (*COI*) is one of the largest protein-coding genes, which functions in electron transport and ATP synthesis and exhibits the most conserved amino acid sequence [37]. It has been widely used in phylogeographic structure and population genetics studies of insects [37], [38], [39]. The Folmer region, 648 base-pairs (bp) at the 5′ end of this gene, has emerged as the standard barcode region [40]. However, few sandfly studies have used this gene [4].

We analyzed the phylogeographic structure of *L. umbratilis* populations on opposite sides of the Negro River and on the left bank of Amazonas River in the central Amazonian Brazil region by analyzing an 1181 bp fragment of the *COI* gene from mtDNA to assess whether *L. umbratilis* populations on both sides comprise incipient or distinct species.

## Results

**Figure 1:**
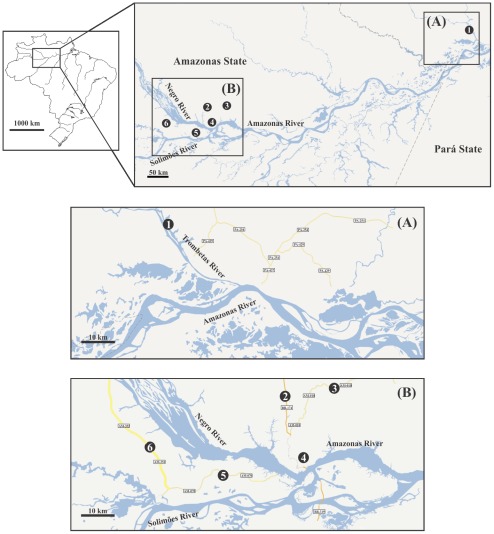
Collection sites of *Lutzomyia umbratilis* from Amazonian Brazil. 1, Cachoeira Porteira; 2, BR-174 Highway; 3, Rio Preto da Eva; 4, Manaus; 5, Manacapuru; 6, Novo Airão. Figures A and B are expanded sizes.

One hundred and eleven *L. umbratilis* specimens were sequenced from six locations from the central Amazonian Brazil region using an 1181 bp fragment of the *COI* gene (Table 1, Figure 1). Nucleotide substitutions were detected in 57 (4.8%) sites, of which 22 (1.86%) were parsimoniously informative. Transitions were more common (93%) than transversions (6.9%), and no deletions or insertions were observed. Base frequencies were estimated as follows: A = 0.3078; T = 0.3991; C = 0.1520; G = 0.1409, where the A+T (∼70.7%) content was similar to that observed for other insects [37].

**Table 1:** Collection sites and sample sizes of *Lutzomyia umbratilis* and *Lutzomyia anduzei* from Amazonian Brazil.

Species	Localities, State	Co-ordinates	N
		Latitude; Longitude	
*L. umbratilis*	Cachoeira Porteira, Oriximiná, Pará	1°28′S; 56°22′W	18
	BR-174 Highway, Amazonas	2°36′S; 60°02′W	15
	Rio Preto da Eva, Amazonas	2°43′S; 59°47′W	15
	Manaus, Amazonas	3°04′S; 59°57′W	4
	Manacapuru, Amazonas	3°14′S; 60°31′W	24
	Novo Airão, Amazonas	2°47′S; 60°55′W	35
*L. anduzei*	Novo Airão, Amazonas	2°47′S; 60°55′W	4
	Autazes, Amazonas	3°42′S; 59°07′W	1
	Amajari, Roraima	3°46′N; 61°44′W	2

N, sample size.

**Figure 2:**
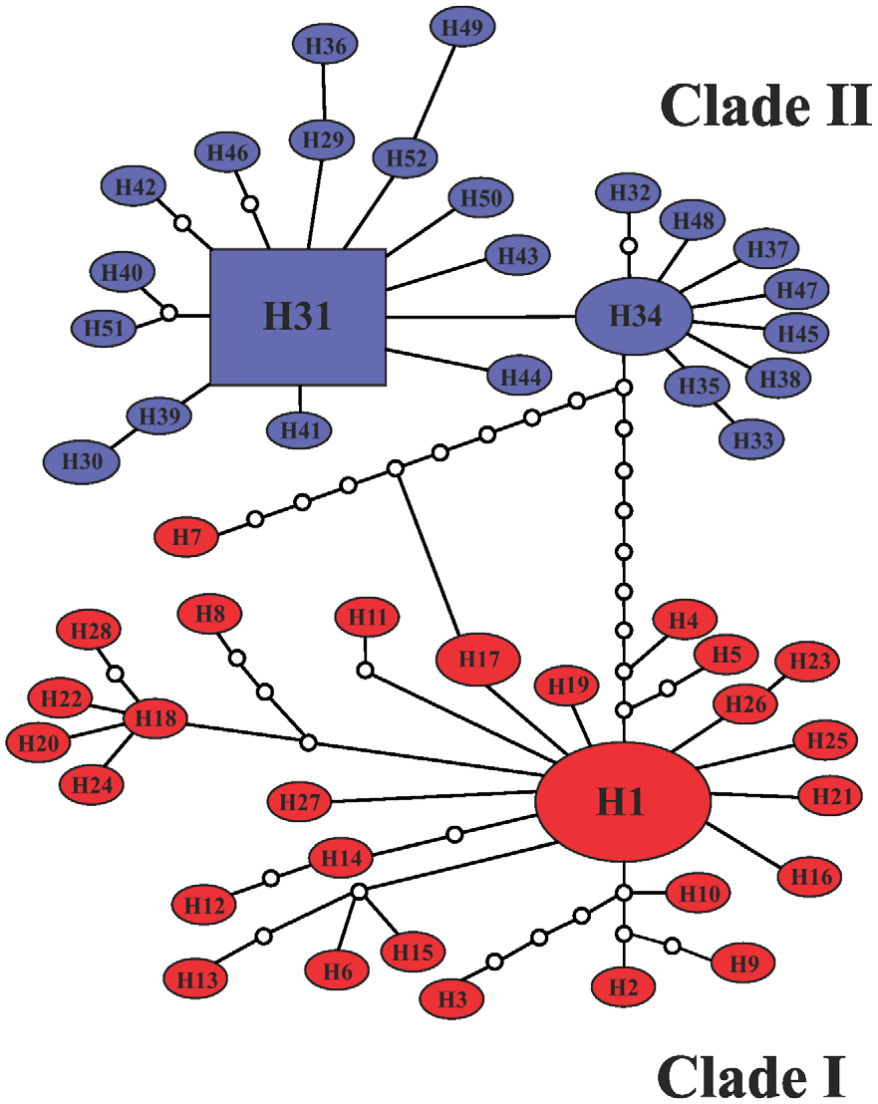
Parsimony haplotypes network of the 52 haplotypes of *Lutzomyia umbratilis*. H1 to H52, haplotypes. The haplotype circle sizes are proportional to number of individuals observed in each haplotype. Empty smaller circles represent mutational events. Clades I and II are in red and blue colors, respectively.

**Figure 3:**
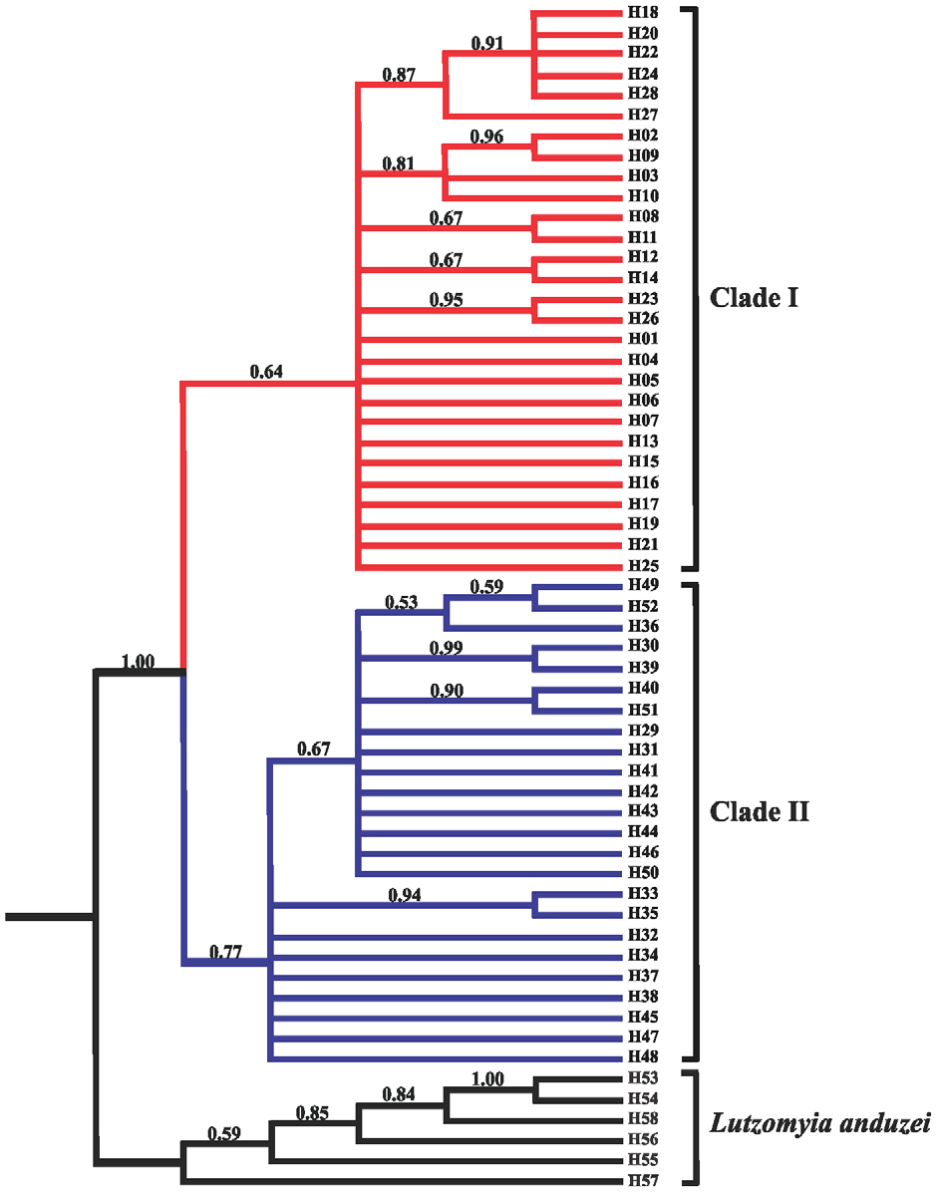
Bayesian Inference (BI) topology tree of the 52 haplotypes of *Lutzomyia umbratilis* inferred under the TIM1+I model. Numbers on each branch (above branch) represent posterior probabilities obtained in the BI. *Lutzomyia anduzei* was used as outgroup.

Table 2 shows the haplotype frequencies in the localities studied. The results revealed a high genetic variation with 52 haplotypes, most of which were singletons and private to their localities (88.46%). The number of haplotypes per locality ranged from four (in Rio Preto da Eva and Manaus) to 17 (in Novo Airão). All haplotypes were connected in the network (Figure 2); however, two distinct haplotype groups ( = clades) separated by ten mutational steps were visualized. Clade I clustered 28 haplotypes (H1 to H28) corresponding to four samples from the left bank of the Negro and Amazonas Rivers: Cachoeira Porteira, 43 km from the BR-174 Highway, Rio Preto da Eva and Manaus (Table 2). Clade II contained 24 haplotypes (H29 to H52) comprising two samples from the right bank of Negro River: Manacapuru and Novo Airão. In clade I, H1 had the highest frequency and it was shared among all samples analyzed from left bank of the Negro and Amazonas Rivers. H17 was shared between the BR-174 Highway and Rio Preto da Eva samples, and based on its position in the haplotypes network may suggest contemporary gene flow. H7 was highly divergent haplotype and formed a reticulation that included H17. Clade II harbored two more frequent haplotypes (H31 and H34) separated by only one mutational event, and H31 may be indicative of older haplotypes. In both clades, the most haplotypes are at the tips and may be more recently derived, characteristic of population expansion [41]. Moreover, four clade I haplotypes (H3, H7, H8, H9 from Cachoeira Porteira) exhibited longer branch-length with missing intermediate. No haplotypes were shared between samples on the opposite banks of rivers. Table S1 shows the variable sites of the haplotypes observed in *L. umbratilis*. The two samples of clade II (H29 to H52) contained five fixed mutations (position sites: 258, 285, 429, 933, 1170). Phylogenetic relationships demonstrated by Bayesian Inference (BI) analysis also revealed two monophyletic clades, but with moderate support (0.64 and 0.77, Figure 3). Clade I clustered the samples from the left bank of the Negro and Amazonas Rivers, whereas the clade II grouped the samples from the right bank of Negro River. Clade II consisted of two subclades (0.67) that resulted of one transition A↔G in the position 660 (Table S1). These substitutions occurred randomly among individuals of the Manacapuru and Novo Airão samples. Six haplotypes (H53 to H58) of outgroup *Lutzomyia anduzei* (Rozeboom, 1942) were strongly supported (1.00) on an isolated cluster. A similar topology tree was visualized by Maximum-Likelihood (ML) analysis (data not shown).

**Table 2:** Haplotype frequency of *Lutzomyia umbratilis* and *Lutzomyia anduzei* from Amazonian Brazil.

Species/Clade	Localities	Haplotype frequency
*L. umbratilis*/Clade I	Cachoeira Porteira	H1(4), H2(1), H3(1), H4(1), H5(1), H6(1),
		H7(1), H8(1), H9(1), H10(1), H11(1),
		H12(1), H13(1), H14(1), H15(1)
	BR-174 Highway	H1(5), H16(1), H17(2), H18(2), H19(1),
		H20(1), H21(1), H22(1), H23(1)
	Rio Preto da Eva	H1(10), H17(3), H24(1), H25(1)
	Manaus	H1(1), H26(1), H27(1), H28(1)
*L. umbratilis*/Clade II	Manacapuru	H29(1), H30(2), H31(8), H32(1), H33(1),
		H34(7), H35(1), H36(1), H37(1), H38(1)
	Novo Airão	H30(2), H31(12), H34(3), H39(5), H40(1),
		H41(1), H42(1), H43(1), H44(1), H45(1),
		H46(1), H47(1), H48(1), H49(1), H50(1),
		H51(1), H52(1)
*L. anduzei*	Novo Airão	H53(1), H54(2), H55(1)
	Autazes	H56(1)
	Amajari	H57(1), H58(1)

H1 to H52, haplotypes of *Lutzomyia umbratilis*; H53 to H58, haplotypes of *Lutzomyia anduzei*. Inside the parentheses is the number of individuals observed for each haplotype. The underlined haplotypes are shared among localities.

The intra-population genetic diversity measures for each sample, combined data and for two clades are shown in Table 3. Haplotype and nucleotide diversities were used as measures of *L. umbratilis* genetic diversity. Within clade I, the Rio Preto da Eva sample exhibited the lowest haplotype and nucleotide diversities, whereas Manaus and Cachoeira Porteira samples had the highest haplotype diversity. Within clade II, two samples showed slightly high haplotype diversity, as did clades I and II. The nucleotide diversity was slightly low in all analyses.

**Table 3:** Intra-population genetic diversity measures for each sample, combined data and haplotype clade of *Lutzomyia umbratilis*.

Samples	Ts/Tv	NS	*K*	*h* ± SE	*π* ± SE
Cachoeira Porteira	27/2	28	4.58	0.961±0.039	0.00389±0.00054
BR-174 Highway	10/0	10	2.02	0.886±0.069	0.00171±0.00029
Rio Preto da Eva	5/0	5	0.88	0.543±0.133	0.00074±0.00030
Manaus	5/0	5	2.67	1.000±0.177	0.00226±0.00073
Manacapuru	9/2	11	1.66	0.819±0.057	0.00141±0.00025
Novo Airão	18/1	19	1.76	0.866±0.048	0.00149±0.00020
Total	54/4	57	6.11	0.926±0.015	0.00517±0.00018
Clade I	37/2	38	2.72	0.848±0.049	0.00231±0.00032
Clade II	24/2	26	1.77	0.853±0.037	0.00150±0.00016

Ts/Tv, transitions/transversions; NS, number of polymorphic sites; *K*, average number of nucleotide differences; *h* ± SE and *π* ± SE, haplotype and nucleotide diversities, respectively, with respective standard errors (SE).

**Figure 4:**
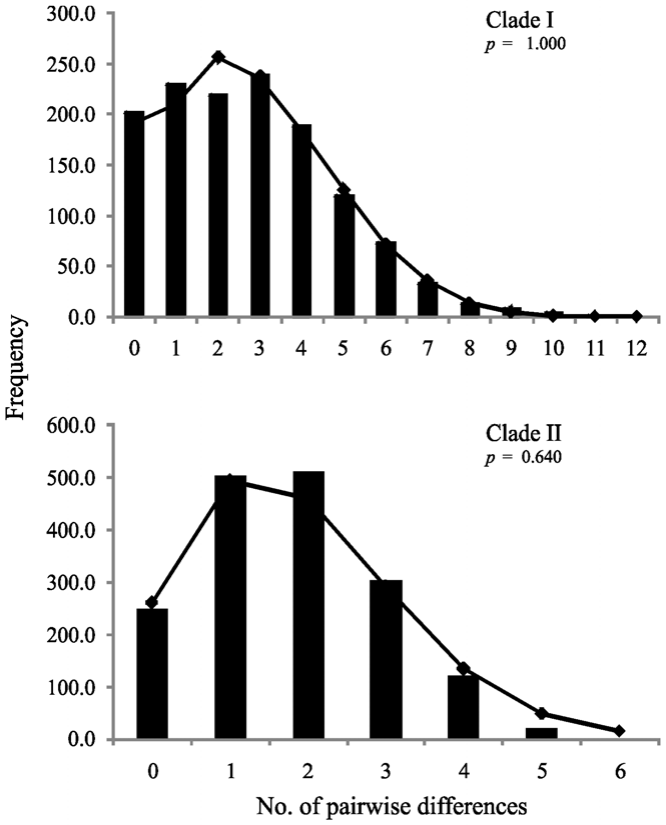
Observed mismatch distributions for the clades I and II of *Lutzomyia umbratilis*. Bars are observed distribution, the line shows the distribution simulated under a sudden expansion model. The *p*-values are from the sum of squared deviations goodness of fit test for the sudden expansion model.

Tajima's *D* test [42] was negative and significant for Cachoeira Porteira and Novo Airão samples (*P*<0.05) and for clades I and II (*P*<0.001), reflecting significant deviations from the neutral model likely due an excess of rare haplotypes consistent with recent population expansion or positive selection (Table 4). Fu's *F*s [43] was negative and significant for Cachoeira Porteira and Novo Airão, combined data and for two clades (*P*<0.0001), and for BR-174 Highway and Manacapuru (*P*<0.05), rejecting the mutation-drift equilibrium and strongly favoring the interpretation of recent population expansion or selective sweeps. Mismatch distribution was unimodal for all samples, except Rio Preto da Eva (data no shown), and for clades I and II (Figure 4), supporting the model of sudden expansion. The bimodal mismatch distribution for Rio Preto da Eva may reflect a bottleneck event. The raggedness index (*r*) calculated for all samples and clades did not reject the null hypothesis of sudden demographic expansion. The sum of squared deviations (*SSD*) goodness-of-fit test was also not significant (Table 4, Figure 4). Thus, the time since the population expansion was calculated for clades I and II using *t* = *τ*/2*μ*, where *μ* is the mutation rate per site per generation [41]. The mutation rate of 10^−8^/site/year [44] and an approximation of five generations/year for *L. umbratilis* [32] were used for this calculation. The time to expansion estimated for clades I and II were approximately 30,448 years ago (95% CI, 12,599–51,947) and 15,859 years ago (95% CI, 11,642–21,566), respectively. Both expansion times were in the late Pleistocene.

**Table 4:** Neutrality tests and population expansion parameters estimated for each sample, combined data and haplotype clade of *Lutzomyia umbratilis*.

Samples	Tajima's *D*	Fu's *F*s	*r*	*SSD*	*τ*
Cachoeira Porteira	−1.833*	−8.620***	0.0357	0.0079	4.404
BR-174 Highway	−1.307	−4.443*	0.0524	0.0045	2.131
Rio Preto da Eva	−1.451	−0.626	0.1391	0.0166	0.732
Manaus	−0.212	−1.414	0.1667	0.0403	1.617
Manacapuru	−1.479	−4.820*	0.0447	0.0006	1.711
Novo Airão	−2.086*	−13.945***	0.0625	0.0030	1.922
Total	−1.409	−34.365***	0.0177	0.0211	10.785
Clade I	−2.312**	−25.421***	0.0073	0.0004	3.596
Clade II	−2.194**	−23.066***	0.0518	0.0015	1.873

*r*, raggedness index; *SSD*, sum of squared deviations; *τ* (tau), expansion parameter.

* , *P*<0.05;

** , *P*<0.001;

*** , *P*<0.0001.

Within clade I, *F*_ST_ values for four samples ranged from low to moderate (−0.0390 to 0.1841) (Table 5). Moreover, the *F*_ST_ value (0.1841) was moderately high between Rio Preto da Eva and Manaus; it likely reflects the small Manaus sample size (n = 4). Similarly, the *F*_ST_ value within clade II between two samples was low (0.0548). Unlikely, the samples between clades I and II yielded very high *F*_ST_ values and were highly significant after the Bonferroni correction (0.7100 to 0.8497). This result was also observed between clades I and II (0.7776).

**Table 5:** Genetic differentiation among samples and haplotype clade of *Lutzomyia umbratilis*.

Samples	*F*_ST_ (Km)	*K*	*D*_xy_	*D*_a_	*S*_s_	*S*_f_
Cachoeira Porteira×BR-174 Highway	0.0522 (368.40)	3.52	0.00297	0.00017	4	0
Cachoeira Porteira×Rio Preto da Eva	0.0569*** (353.67)	2.99	0.00248	0.00017	2	0
Cachoeira Porteira×Manaus	0.0230 (394.02)	3.92	0.00332	0.00025	1	0
BR-174 Highway×Rio Preto da Eva	0.0189 (30.46)	1.48	0.00125	0.00002	3	0
BR-174 Highway×Manaus	−0.0390 (56.29)	2.20	0.00187	−0.00012	3	0
Rio Preto da Eva×Manaus	0.1841 (45.35)	1.87	0.00158	0.00008	2	0
Manacapuru×Novo Airão	0.0548 (58.74)	1.81	0.00153	0.00008	4	0
Cachoeira Porteira×**Manacapuru**	**0.7100** *** (449.22)	10.19	**0.00863**	**0.00599**	**2**	**3**
BR-174 Highway×**Manacapuru**	**0.8157** *** (87.11)	9.78	**0.00833**	**0.00673**	**0**	**6**
Rio Preto da Eva×**Manacapuru**	**0.8497** *** (96.01)	8.98	**0.00765**	**0.00653**	**0**	**6**
Manaus×**Manacapuru**	**0.8249** *** (59.43)	10.42	**0.00887**	**0.00699**	**0**	**7**
Cachoeira Porteira×**Novo Airão**	**0.7337** *** (477.49)	10.55	**0.00899**	**0.00625**	**6**	**1**
BR-174 Highway×**Novo Airão**	**0.8197** *** (107.97)	10.21	**0.00869**	**0.00705**	**4**	**4**
Rio Preto da Eva×**Novo Airão**	**0.8439** *** (130.14)	9.43	**0.00803**	**0.00687**	**1**	**4**
Manaus×**Novo Airão**	**0.8269** *** (108.76)	10.84	**0.00924**	**0.00731**	**2**	**5**
**Clade I**×**Clade II**	**0.7776** ***	9.99	**0.00850**	**0.00660**	**8**	**1**
*L. umbratilis*×*L. anduzei*		12.78	0.05747	0.04870	4	43

*F*_ST_, pair-wise genetic differentiation; *K*, average number of nucleotide differences between populations; *D*_xy_, average number of nucleotide substitutions per site between populations; *D*_a_, number of net nucleotide substitutions per site between populations; *S*_s_, number of shared polymorphisms between pairs of populations; *S*_f_, number of fixed differences between pairs of populations. The geographic distance (in km) between localities is represented inside the parentheses.

*** *P* = 0.00000±0.0000, after the Bonferroni correction.

Table 5 also shows the average number of nucleotide substitution per site between populations (*D_xy_*), the number of net nucleotide substitutions per site between populations (*D_a_*), the number of shared polymorphisms (*S_s_*) and the number of fixed differences (*S_f_*). Similarly, *F*_ST_, the highest *D_xy_* and *D_a_* values were detected between clades I and II. Furthermore, few or no shared polymorphisms or fixed differences were found between samples of two clades, particularly in comparisons between Manacapuru and three BR-174 Highway, Rio Preto da Eva and Manaus samples (*S_s_* = 0; *S_f_* = 6–7). Interestingly, Manacapuru versus Cachoeira Porteira and Novo Airão versus Cachoeira Porteira, which are geographically further apart, harbored more shared polymorphisms and less fixed differences (*S_s_* = 2, *S_f_* = 3; *S_s_* = 6, *S_f_* = 1, respectively). Although the *F*_ST_ value (0.0569) was statistically significant between Cachoeira Porteira and Rio Preto da Eva (both from clade I), the *D_xy_* and *D_a_* values were low, and no fixed differences were found between them. As described above, the genetic divergence observed between the populations does not fit the isolation-by-distance model. Analyzing all samples together, the correlation between genetic and geographic distances was negative (*r* = −0.063116) and not significant (*P* = 0.366), perhaps because geographically closer populations are more differentiated, which likely can be attributed to the rivers acting as barriers.

Sequence divergence and the average number of nucleotide differences (*K*) between clades I and II were 1.0% and 9.99, respectively, whereas between *L. umbratilis* and the closely related species *L. anduzei* were ∼5.8% and 12.78, respectively. Given an evolutionary rate of 2.3% per million years estimated for the *COI* gene in insects [45], the divergence time suggests that the two clades have diverged approximately 0.22 Mya during the middle Pleistocene.

## Discussion

### Genetic diversity and phylogeographic structure

In this study, the phylogenetic analyses revealed the presence of two distinct *L. umbratilis* clades from the opposite sides of the rivers, which were separated by a substantial genetic division in the haplotype network. Similarly, a high and significant genetic differentiation (*F*_ST_ = 0.7100–0.8497) was detected between two clades. These results combined with the lack of shared haplotypes and presence of fixed differences between populations of these clades suggest that the Amazonas and Negro Rivers may be acting as effective barriers, thus preventing gene flow between *L. umbratilis* populations from opposite sides.

The genetic differentiation detected in this study supports the biological and morphological differences initially observed by Justianiano *et al.* [32] and Justianiano [33]. Taken together, these findings might explain possible differences in the vector competence of these sandflies, a hypothesis proposed by Arias and Freitas [25]. Further studies of experimental infections and transmission dynamics (anthropophily degree, resting behavior, biting peak, infectivity rate of samples from field) in these populations are needed to confirm or refute this hypothesis.

The *F*_ST_ values detected between samples of two clades were similar to those described by Soto *et al.* [16] between samples of *Lutzomyia longipalpis* Lutz & Neiva, 1912 from Central and South America using polymorphisms of the *ND4* gene from mtDNA (*F*_ST_ = 0.52 to 0.933), over a much larger geographic area than in the present study. Another study analyzed nine populations of the *L. longipalpis* complex from northeastern region and state of Minas Gerais, Brazil, using *Ctyb* gene sequences from mtDNA and a larger geographic area [46]. They also detected the presence of two groups but a lower genetic differentiation (*F*_ST_ = 0.184) than that observed here. Comparing these findings, our very high *F*_ST_ values over short geographic distances appear rare in sandflies species, and according to Wright [47] this genetic differentiation is fairly high. Additionally, the divergence degree detected between two lineages of the present study fall within a differentiation range described between two cryptic species of *Anopheles cruzii* complex [1], thus suggesting that two species could exist within *L. umbratilis*, although this hypothesis was moderately supported in the phylogenetic analyses.

Unlikely, the genetic differentiation verified among *L. umbratilis* samples within each clade (samples from the same side of the rivers) was low, even when populations were separated by geographic distances ranging 353.67 to 394.02 km apart (Table 5), a differentiation degree consistent with those described for populations within species [47]. This result may be due to ongoing gene flow or sharing of a recent common history, as recent population expansion (Table 4, Figures 2 and 4). Taken together, this study suggests that *L. umbratilis* populations across rivers from the central Amazonian Brazil region may represent an advanced speciation process consisting of two incipient or distinct species that likely initiated their independent evolutionary histories after the complete formation of the Amazonas and Negro Rivers between the late Tertiary and early Quaternary ∼5.3 to 1.6 Mya [48].

### Riverine Barrier Hypothesis

The riverine barrier hypothesis originally proposed by Wallace [49] states that major Amazonian rivers significantly reduce or prevent gene flow between populations inhabiting opposite river banks, promoting speciation. In a phylogenetic and population genetics approach, the three main predictions of the riverine barrier hypothesis are as follows: (1) sister intraspecific clades and species will occur across major rivers rather than within major Amazonian interfluves [50], [51]; (2) phylogeographic analyses should allow the differentiation of primary divergence across rivers (predicted by the riverine barrier hypothesis) from secondary contact along rivers between nonsister lineages [50], [51]; and (3) within a river basin, genetic similarity between populations separated by a river should be higher in the headwaters, where the river is narrower, than in its lowest part [51], [52].

The first prediction fits the best with our data. The two genetic lineages observed in this study may be derived from an ancestral gene pool, and their complete fragmentation would have occurred after the more recent formation of the Amazonas and Negro Rivers, ∼2.4 Mya to present [53], attributed as the most probable evolutionary force. This vicariant event allied the low dispersal rate of the sandflies, and the amenable environmental conditions for adaptation and also drift should have contributed to the high divergence level between the populations of these banks. The deep genetic split in these populations across rivers is also supported by other facts. The lack of isolation by distance among samples and the strong bimodality observed in the mismatch distribution when all samples were analyzed together (data not shown) are consistent with long-term isolation and the lack of recent gene flow between these populations. Avise [54] and He *et al.* [55] have argued that significant evolutionary clustering of haplotypes in different geographical regions has been frequently interpreted as evidence of a past population fragmentation, particularly when haplotype clusters are separated by long branch-length with missing intermediates. This argument fits the framework of the *L. umbratilis* populations on opposite banks, as represented in the Figure 2.

However, the estimated sequence divergence between two lineages was lower (1.0%) that between two closely related species *L. umbratilis* and *L. anduzei* (5.8%) of this study and among four clades of *L. longipalpis* complex (9.47–10.91%) using *COI* gene [4]. A rough estimate of divergence time suggests that the separation between *L. umbratilis* populations from opposite banks was ∼0.22 Mya during the middle Pleistocene. Although this comparison involves different genetic markers, this divergence time suggests that *L. umbratilis* diversification from the central Amazonian region may be more recent than that estimated for the *L. longipalpis* complex based on two genes (*Cacophony* IVS6 Intron gene = 0.98 to 1.47 Mya [56]; *Cytb* gene = 0.45 Mya [46]). Based on the 1–2.5% divergence with the *Cytb* gene, Esseghir *et al.* [57] proposed that diversification among closely related species in the *Phlebotomus* genus may have occurred in the Quaternary period, and our findings support this hypothesis. On the other hand, this separation time may be insufficient for two lineages of *L. umbratilis* to have formed distinct species, based on the allopatric speciation studies in *Drosophila* [58]. Nonetheless, the hypothesis that these lineages could represent two species will only be confirmed or refuted with further studies using other molecular genetic markers and additional sampling along the river banks and within interfluves.

### Recent expansion of *L. umbratilis* populations

All samples, except Rio Preto da Eva, and the two clades analyzed in this study exhibited high levels of genetic diversity measured by the haplotype diversity. In contrast, the nucleotide diversity was slightly low when compared to other Diptera species [3], [38], [59]. This pattern of genetic diversity may reflect population expansion after a period of low effective population size likely caused by bottlenecks or founder events where populations contracted. The star-shaped haplotype network detected for *L. umbratilis* is also consistent with population expansion (Figure 2), where the most haplotypes are at the tips and, in such cases, the sudden expansion enhances the retention of new mutations [43]. Additionally, Tajima's *D* and Fu's *F*s tests were negative and highly significant for two lineages and for samples from Cachoeira Porteira and Novo Airão, as well as the mismatch distribution was unimodal. Taken together, these data strongly suggest that a recent population expansion attributed to the deviation from neutral expectations in these populations rather than background selection. The estimate time of expansion for the lineages I and II was ∼30,448 and 15,859 years ago, respectively. The confidence intervals of these estimates overlap, suggesting that these expansions could have occurred at the same time in the late Pleistocene. The climatic conditions and presence of animal and human populations likely created more favorable habitats for expansions of these populations.

The two genetic lineages detected in this study that may also differ biologically [32] and morphologically [33] are of vital importance for epidemiological studies, especially those related to vector competence, anthropophily and behavioral aspects, and for vector control strategies, including the genetic control. In addition, *L. umbratilis* represents an interesting example in speciation studies.

## Materials and Methods

### Sandfly collection and identification

*Lutzomyia umbratilis* adults were sampled from six localities including Cachoeira Porteira, in the municipality of Oriximiná, in Pará, Brazil; at km 43 of BR-174 Highway and km 65 of AM-010 Highway in the municipality of Rio Preto da Eva, Manaus, km 60 of AM-070 Highway in the municipality of Manacapuru, and km 60 and km 70 of AM-352 Highway in the municipality of Novo Airão, in Amazonas, Brazil (Figure 1). Table 1 summarizes the collection sites, co-ordinates, and the sample sizes. Sandflies were collected with CDC (Centers for Disease Control) miniature light traps and with aspirators on the bases of tree trunks. To collect a randomly population sample and avoid an excess of offspring from the same females, we sampled more than five tree trunks within the same area. Both sides of Highways and roads within the collection area were sampled. The specimens were preserved in 95% ethanol and stored at −80°C until total DNA was extracted. Morphological identification was performed on genitalia according to the taxonomic key of Young and Duncan [11]. This study and catch protocol was reviewed and approved by the Institutional Review Board of the National Institute of Research of the Amazon, the Brazilian Ministry of Science, Technology and Innovation, Brazil. The sample collections were authorized by the Brazilian Institute of Environment and Natural Renewable Resources (IBAMA) and by the Authorization System and Information on Biodiversity (SISBIO), license number 12733-1 for the collections of *L. umbratilis* and *L. anduzei* from the states of Amazonas and Roraima, Brazil, and license number 14054-5 for the collections of *L. umbratilis* from Cachoeira Porteira, state of Pará, Brazil.

### DNA extraction, PCR, and sequencing

Total genomic DNA was extracted individually from whole sandflies using a phenol and chloroform method [60], resuspended in 20 µL of 1× TE buffer (10 mM Tris-Cl pH 8.0, 1 mM EDTA pH 8.0) and stored at −80°C until PCR (Polymerase Chain Reaction) analysis. An 1181 bp fragment from the *COI* gene was amplified using 10 µM primers UEA3/UEA10. The primer sequences and amplification conditions are described in Zhang and Hewitt [61]. The PCR products were visualized on 1% agarose gels under UV light, purified with PEG and sequenced bi-directionally on an Automated Sequencer (ABI 3130 XL model, Applied Biosystems).

### Phylogenetic analysis

The sequences were edited by using the BIOEDIT [62]. The estimated genealogical relationships of the haplotypes were generated in the TCS, 1.21 [63], at the 95% confidence level and the homoplasies were resolved using the rules of Crandall and Templeton [64]. Phylogenetic relationships among the haplotypes were inferred by Bayesian inference (BI) analysis, which was implemented in the MR. BAYES [65], using the evolutionary model (TIM1+I) that best fit the *COI* data sets; this model was previously determined in the jMODELTEST [66]. The settings were two simultaneous independents runs of the Markov Chain Monte Carlo (MCMC) for 100 million generations, sampling every 1,000 generations with a burn in of 25%. *Lutzomyia anduzei* was selected as an outgroup in the phylogenetic analysis based on the morphologic similarities to *L. umbratilis* [11], which have been misidentified as *L. anduzei* in the past [12]. Both species have a large overlapping region in northern South America [11]. *Lutzomyia anduzei* is also an anthropophilic species [11] and can be a secondary vector of *Le. guyanensis* [18]. In the present study, four *L. anduzei* specimens from municipality of Novo Airão (Amazonas, Brazil), one from municipality of Autazes (Amazonas, Brazil), and two from Amajari (Roraima, Brazil) were sequenced as shown in the Tables 1 and 2. These sequences (H53 to H58) were used as outgroup in the phylogenetic analyses (Figure 3).

### Genetic diversity and population structure

The intra-population genetic diversity measures (haplotype and nucleotide diversities, *K* values, number of polymorphic sites, transition and transversion rates) and Tajima's *D* [42] and Fu's *F*s tests [43] for each sample, combined data and for the two clades were inferred using DNASP, 4.0 [67] and ARLEQUIN, 3.1 [68]. A neutrality test, Tajima's *D*, was implemented to test strict neutrality, whereas the neutrality test, Fu's *F*s, was estimated to test population size stability. The latter test is more powerful for detecting population expansion and genetic hitchhiking. The mismatch distribution (an observed distribution of pair-wise nucleotide differences among haplotypes) was computed for each sample, combined data and for clades I and II in ARLEQUIN, 3.1 [68] with 1,000 permutations. The raggedness index (*r*) [69] and sum of squared deviations (*SSD*) between observed and expected mismatch distributions were calculated as a test statistic to validate the estimated sudden expansion model. Genetic differentiation, on the basis of *F*_ST_ pairwise between samples and between clades, was inferred in ARLEQUIN, 3.1 [68], whereas the average number of nucleotide substitutions per site between populations (*D_xy_*) [70], the number of net nucleotide substitutions per site between populations (*D_a_*), the number of shared polymorphisms between population pairs (*S_s_*) and number of fixed differences between population pairs (*S_f_*) were calculated in DNASP, 4.0 [67].

The estimated divergence time between the two clades was calculated assuming a mutation rate of 2.3% per million years for the *COI* gene [45]. This calculation used the sequence divergence between the two clades based on the Kimura two-parameter (K2P) evolutionary model estimated in MEGA, 4.0 [71]. The sequence divergence between *L. umbratilis* and the *L. anduzei* outgroup was also estimated based on the K2P.

The correlation between straight-line geographic distances and *F*_ST_ values among samples was assessed using Mantel test [72] in ARLEQUIN, 3.1 [68] with 1,000 permutations. Straight-line geographic distances were estimated in Google Earth. Haplotypes sequences of *L. umbratilis* (H1, H30, H31) and *L. anduzei* (H53, H54, H55) are deposited in GenBank under the accession numbers JQ839256 to JQ839261.

## Supporting Information

Table S1 **Variable sites of haplotypes of** ***Lutzomyia umbratilis*** **and** ***Lutzomyia anduzei*** **.** H, haplotypes; H1 to H52, haplotypes of *Lutzomyia umbratilis*; H53 to H58, haplotypes of *Lutzomyia anduzei*. The dots indicate identical nucleotides with the H1. The frequency of each haplotype is provided in Table 2.
(DOC)
