# Supplementary material for: Lutzomyia umbratilis, the Main Vector of Leishmania guyanensis, Represents a Novel Species Complex?
Source: PLoS One. 2012 May 18;7(5):e37341. doi: 10.1371/journal.pone.0037341 (PMC3356248; doi:10.1371/journal.pone.0037341)
Supplement: Table S1 — Variable sites of haplotypes of Lutzomyia umbratilis and Lutzomyia anduzei . H, haplotypes; H1 to H52, haplotypes of Lutzomyia umbratilis; H53 to H58, haplotypes of Lutzomyia anduzei. The dots indicate identical nucleotides with the H1. The frequency of each haplotype is provided in Table 2. (DOC) [file pone.0037341.s001.doc]

|  |  |  |  |  |  |  |  |  |  | 1 | 1 | 1 | 1 | 1 | 1 | 2 | 2 | 2 | 2 | 2 | 2 | 2 | 2 | 2 | 2 | 3 | 3 | 3 | 3 | 3 | 3 | 3 | 3 | 3 | 3 | 3 | 3 | 3 | 3 | 4 | 4 | 4 | 4 | 4 | 4 | 4 | 4 | 4 | 5 | 5 | 5 | 5 | 5 | 5 | 5 | 5 |
| --- | --- | --- | --- | --- | --- | --- | --- | --- | --- | --- | --- | --- | --- | --- | --- | --- | --- | --- | --- | --- | --- | --- | --- | --- | --- | --- | --- | --- | --- | --- | --- | --- | --- | --- | --- | --- | --- | --- | --- | --- | --- | --- | --- | --- | --- | --- | --- | --- | --- | --- | --- | --- | --- | --- | --- | --- |
| H |  | 2 | 3 | 4 | 6 | 6 | 7 | 8 | 8 | 2 | 7 | 7 | 7 | 9 | 9 | 1 | 1 | 4 | 5 | 5 | 5 | 6 | 7 | 8 | 9 | 2 | 3 | 3 | 4 | 4 | 5 | 5 | 6 | 6 | 7 | 8 | 9 | 9 | 9 | 0 | 2 | 2 | 3 | 4 | 6 | 6 | 7 | 8 | 0 | 1 | 2 | 2 | 3 | 4 | 6 | 6 |
|  | 7 | 7 | 6 | 2 | 0 | 3 | 9 | 4 | 7 | 0 | 1 | 5 | 7 | 2 | 5 | 6 | 9 | 6 | 5 | 6 | 8 | 1 | 6 | 5 | 8 | 1 | 0 | 6 | 2 | 5 | 1 | 4 | 0 | 6 | 8 | 4 | 0 | 3 | 6 | 2 | 0 | 9 | 5 | 7 | 2 | 8 | 7 | 9 | 5 | 9 | 0 | 5 | 4 | 6 | 1 | 7 |
| H1 | T | A | A | A | T | C | A | T | T | A | T | G | A | T | A | A | T | A | A | C | G | C | A | G | C | C | C | T | A | T | A | C | T | C | T | T | T | T | T | C | G | G | T | T | A | A | C | A | T | A | C | A | T | C | T | T |
| H2 | . | . | . | . | . | . | . | . | . | . | . | . | . | . | . | . | . | . | . | . | . | . | . | A | . | . | . | . | . | . | . | . | . | . | . | . | . | . | . | . | . | . | . | . | . | . | . | . | . | . | . | . | . | . | . | . |
| H3 | . | . | . | . | . | . | . | . | . | . | . | . | . | . | . | . | . | . | . | . | . | . | . | A | . | . | . | . | . | . | . | . | . | . | . | . | . | . | . | . | . | . | . | . | G | . | . | . | . | . | . | . | . | . | . | . |
| H4 | . | . | . | . | . | . | . | . | . | . | . | . | . | . | . | G | . | . | . | . | . | . | . | . | . | . | . | . | . | . | . | . | . | . | . | . | . | . | . | . | . | A | . | . | . | . | . | . | . | . | . | . | . | . | . | . |
| H5 | . | . | . | . | . | . | . | . | . | . | . | . | . | . | . | . | . | . | . | . | . | . | . | . | . | . | . | . | . | . | . | . | . | . | . | . | . | . | . | . | A | A | . | . | . | . | . | . | . | . | . | . | . | . | . | . |
| H6 | . | . | G | . | . | . | . | . | . | . | . | . | . | . | . | . | . | . | . | . | . | . | . | . | . | T | . | . | . | . | . | . | . | . | . | . | . | . | . | . | . | . | . | . | . | . | . | . | . | . | . | . | . | . | . | . |
| H7 | . | . | . | . | . | . | . | . | . | . | . | . | . | . | . | . | . | . | . | . | A | . | . | . | . | T | . | . | . | . | . | . | . | . | . | C | . | . | . | . | . | A | . | . | . | . | . | . | . | G | . | . | . | . | . | . |
| H8 | . | . | . | . | . | . | . | . | . | . | . | . | . | . | . | . | . | . | . | . | . | . | . | . | . | . | T | . | . | . | . | T | . | . | . | . | . | . | . | . | . | . | . | . | . | . | . | . | . | . | . | . | . | . | . | . |
| H9 | . | . | . | . | . | . | . | . | . | . | . | . | . | . | . | . | . | . | G | . | . | . | . | A | . | . | . | . | . | . | . | . | . | . | . | . | C | . | . | . | . | . | . | . | . | . | . | . | . | . | . | . | . | . | . | . |
| H10 | . | . | . | . | . | . | . | . | . | . | . | . | . | . | . | . | . | . | . | . | . | . | . | A | . | . | . | . | . | . | . | . | . | . | . | . | . | . | . | . | . | . | . | . | . | . | . | . | . | . | . | . | . | . | . | . |
| H11 | . | . | . | . | . | . | . | . | . | . | . | . | . | . | . | . | . | . | . | . | . | . | . | . | . | . | . | . | . | . | . | . | . | . | . | . | . | . | . | . | . | . | . | . | . | . | . | . | . | . | . | . | . | . | . | . |
| H12 | T | . | . | . | . | . | . | . | . | . | . | . | . | . | . | . | . | . | . | . | . | . | . | . | . | T | . | . | . | . | . | T | . | . | . | . | . | . | . | . | . | . | . | . | . | . | . | . | . | . | . | . | . | . | . | . |
| H13 | . | . | . | . | . | . | . | . | . | . | . | . | . | . | . | . | . | . | . | . | . | . | . | . | . | T | . | . | . | . | . | . | . | . | . | . | . | . | . | . | A | . | . | . | . | . | . | . | . | . | . | . | . | . | . | . |
| H14 | . | . | . | . | . | . | . | . | . | . | . | . | . | . | . | . | . | . | . | . | . | . | . | . | . | T | . | . | . | . | . | T | . | . | . | . | . | . | . | . | . | . | . | . | . | . | . | . | . | . | . | . | . | . | . | . |
| H15 | . | . | . | . | . | . | . | . | . | . | . | . | . | . | . | . | C | . | . | . | . | . | . | . | . | T | . | . | . | . | . | . | . | . | . | . | . | . | . | . | . | . | . | . | . | . | . | . | . | . | . | . | . | . | . | . |
| H16 | . | . | . | . | . | . | . | . | . | . | . | . | . | . | . | . | . | . | . | . | . | . | . | . | . | . | . | . | . | . | . | . | . | . | . | . | . | . | . | . | . | . | . | . | . | . | . | . | . | . | . | . | . | T | . | . |
| H17 | . | . | . | . | . | . | . | . | . | . | . | . | . | . | . | . | . | . | . | . | A | . | . | . | . | . | . | . | . | . | . | . | . | . | . | . | . | . | . | . | . | . | . | . | . | . | . | . | . | . | . | . | . | . | . | . |
| H18 | . | . | . | . | . | . | . | . | . | . | . | . | . | . | . | . | . | . | . | . | . | . | . | . | . | . | . | . | . | . | . | T | . | . | . | . | . | . | . | . | . | . | . | C | . | . | . | . | . | . | . | . | . | . | . | . |
| H19 | . | . | . | . | . | . | . | . | . | . | . | A | . | . | . | . | . | . | . | . | . | . | . | . | . | . | . | . | . | . | . | . | . | . | . | . | . | . | . | . | . | . | . | . | . | . | . | . | . | . | . | . | . | . | . | . |
| H20 | . | . | . | . | . | . | . | . | . | . | . | . | . | . | . | . | . | . | . | . | . | . | . | . | . | . | . | . | . | . | . | T | . | . | . | . | . | . | . | . | . | . | . | C | . | . | . | . | . | . | . | . | . | . | . | . |
| H21 | . | . | . | . | C | . | . | . | . | . | . | . | . | . | . | . | . | . | . | . | . | . | . | . | . | . | . | . | . | . | . | . | . | . | . | . | . | . | . | . | . | . | . | . | . | . | . | . | . | . | . | . | . | . | . | . |
| H22 | . | . | . | . | . | . | . | . | . | . | . | . | . | . | . | . | . | . | . | . | . | . | . | . | . | . | . | . | . | . | . | T | . | . | . | . | . | . | . | . | . | . | . | C | . | . | . | . | . | . | . | . | . | . | . | . |
| H23 | . | . | . | . | . | . | . | . | . | . | . | . | . | . | . | . | . | . | . | . | . | . | . | . | . | . | . | . | . | . | . | . | . | . | . | . | . | . | . | . | . | . | . | . | . | . | . | . | . | . | . | . | . | . | . | . |
| H24 | . | . | . | . | . | . | . | . | . | . | . | . | . | . | . | . | . | . | . | . | . | . | . | . | . | . | . | . | . | . | . | T | . | . | . | . | . | . | . | . | . | . | . | C | . | . | . | . | . | . | . | . | . | . | . | . |
| H25 | . | . | . | . | . | . | G | . | . | . | . | . | . | . | . | . | . | . | . | . | . | . | . | . | . | . | . | . | . | . | . | . | . | . | . | . | . | . | . | . | . | . | . | . | . | . | . | . | . | . | . | . | . | . | . | . |
| H26 | . | . | . | . | . | . | . | . | . | . | . | . | . | . | . | . | . | . | . | . | . | . | . | . | . | . | . | . | . | . | . | . | . | . | . | . | . | . | . | . | . | . | . | . | . | . | . | . | . | . | . | . | . | . | . | . |
| H27 | . | . | . | . | . | . | . | . | . | . | . | . | . | . | . | . | . | . | . | . | . | . | . | . | . | . | . | . | . | . | . | . | . | . | . | . | . | . | . | . | . | . | . | C | . | . | . | . | . | . | . | . | . | . | . | T |
| H28 | . | . | . | . | . | . | . | . | . | . | . | . | . | . | . | . | . | . | . | . | . | T | . | . | . | . | . | . | . | . | . | T | . | . | . | . | T | . | . | . | . | . | . | C | . | . | C | . | . | . | . | . | . | . | . | . |
| H29 | . | . | . | . | . | . | . | . | . | . | . | . | . | . | . | . | . | . | . | . | A | . | . | A | . | . | . | . | G | . | . | . | . | . | . | . | . | . | T | . | . | A | . | . | . | . | . | . | . | . | . | . | . | . | . | . |
| H30 | . | T | . | . | . | . | . | . | . | . | . | . | . | . | . | . | . | . | . | . | A | . | . | A | . | . | . | . | . | . | . | . | . | . | . | . | . | . | . | . | . | A | . | . | . | . | . | . | . | . | . | . | . | . | . | . |
| H31 | . | . | . | . | . | . | . | . | . | . | . | . | . | . | . | . | . | . | . | . | A | . | . | A | . | . | . | . | G | . | . | . | . | . | . | . | . | . | T | . | . | A | . | . | . | . | . | . | . | . | . | . | . | . | . | . |
| H32 | . | . | . | . | . | . | . | . | . | . | . | . | . | . | . | . | . | . | . | . | A | . | . | A | . | . | . | . | G | . | . | . | . | . | . | . | . | . | . | . | . | A | . | . | . | . | . | . | . | . | . | . | . | . | . | . |
| H33 | . | . | . | . | . | . | . | . | . | . | C | . | . | A | . | . | . | . | . | . | A | . | . | A | . | . | . | . | G | . | . | . | . | . | . | . | . | . | . | . | . | A | . | . | . | . | . | . | . | . | . | . | . | . | . | . |
| H34 | . | . | . | . | . | . | . | . | . | . | . | . | . | . | . | . | . | . | . | . | A | . | . | A | . | . | . | . | G | . | . | . | . | . | . | . | . | . | . | . | . | A | . | . | . | . | . | . | . | . | . | . | . | . | . | . |
| H35 | . | . | . | . | . | . | . | . | . | . | C | . | . | . | . | . | . | . | . | . | A | . | . | A | . | . | . | . | G | . | . | . | . | . | . | . | . | . | . | . | . | A | . | . | . | . | . | . | . | . | . | . | . | . | . | . |
| H36 | . | . | . | . | . | . | . | . | . | . | . | . | . | . | . | . | . | . | . | . | A | . | . | A | . | T | . | . | G | . | . | . | . | . | . | . | . | . | . | . | . | A | . | . | . | . | . | . | . | . | . | . | . | . | . | . |
| H37 | . | . | . | . | . | . | . | . | . | . | . | . | . | . | . | . | . | . | . | . | A | . | . | A | . | . | . | . | G | . | . | . | . | . | . | . | . | . | . | . | . | A | . | . | . | . | . | . | . | . | . | . | . | . | . | . |
| H38 | . | . | . | . | . | . | . | . | . | . | . | . | . | . | . | . | . | . | . | . | A | . | . | A | . | . | . | . | G | . | . | . | . | . | . | . | . | . | . | . | . | A | . | . | . | . | . | . | . | . | . | G | . | . | T | . |
| H39 | . | T | . | . | . | . | . | . | . | . | . | . | . | . | . | . | . | . | . | . | A | . | . | A | . | . | . | . | G | . | . | . | . | . | . | . | . | . | . | . | . | A | . | . | . | . | . | . | . | . | . | . | . | . | . | . |
| H40 | . | . | . | . | . | . | . | . | . | . | . | . | . | . | . | . | . | . | . | . | A | . | . | A | . | . | . | . | G | . | . | . | . | . | . | . | . | . | . | . | . | A | . | . | . | . | . | . | . | . | . | . | . | . | . | . |
| H41 | . | . | . | . | . | . | . | . | . | . | . | . | . | . | . | . | . | . | . | . | A | . | . | A | . | . | . | . | G | . | . | T | . | . | . | . | . | . | . | . | . | A | . | . | . | . | . | . | . | . | . | . | . | . | . | . |
| H42 | . | . | . | . | . | . | . | . | . | . | . | . | . | . | . | . | . | . | . | . | A | . | . | A | . | . | . | . | G | . | . | . | . | . | . | . | . | . | . | . | . | A | . | . | . | . | . | . | . | . | . | . | . | . | . | . |
| H43 | . | . | . | . | . | . | . | . | . | . | . | . | . | . | . | . | . | . | . | . | A | . | . | A | . | . | . | . | G | . | . | . | . | . | . | . | . | . | . | . | . | A | . | . | . | . | . | . | . | . | . | . | . | . | . | . |
| H44 | . | . | . | . | . | . | . | . | . | . | . | . | . | . | . | . | . | . | . | . | A | . | . | A | . | . | . | . | G | . | . | . | . | . | . | . | . | . | . | . | . | A | . | . | . | . | . | . | . | . | . | . | . | . | . | . |
| H45 | . | . | . | . | . | . | . | . | . | . | . | . | . | . | . | . | . | . | . | . | A | . | . | A | . | . | . | . | G | . | . | . | . | . | . | . | . | . | . | . | . | A | . | . | G | . | . | . | . | . | . | . | . | . | . | . |
| H46 | C | . | . | . | . | . | . | . | . | . | . | . | . | . | . | . | . | . | . | . | A | . | . | A | . | . | . | . | G | . | . | . | . | . | . | . | . | . | . | . | . | A | . | . | . | . | . | . | . | . | . | . | . | . | . | . |
| H47 | . | . | . | . | . | . | . | C | . | . | . | . | . | . | . | . | . | . | . | . | A | . | . | A | . | . | . | . | G | . | . | . | . | . | . | . | . | . | . | . | . | A | . | . | . | . | . | . | . | . | . | . | . | . | . | . |
| H48 | . | . | . | . | . | . | . | . | . | . | . | . | . | . | . | . | . | . | . | . | A | . | . | A | . | . | . | . | G | . | . | . | . | . | . | . | . | . | . | . | . | A | . | . | . | . | . | . | . | . | . | . | . | . | . | . |
| H49 | . | . | . | . | . | . | . | . | . | . | . | . | . | . | . | . | . | . | . | . | A | . | . | A | . | T | . | . | G | T | . | . | . | . | . | . | . | . | . | . | . | A | . | . | . | . | . | . | . | . | . | . | . | . | . | . |
| H50 | . | . | . | . | . | . | . | . | . | . | . | . | . | . | . | . | . | . | . | . | A | . | . | A | . | . | . | . | G | . | . | . | . | . | . | . | . | . | . | . | . | A | C | . | . | . | . | . | . | . | . | . | . | . | . | . |
| H51 | . | . | . | . | . | . | . | . | . | . | . | . | . | . | . | . | . | . | . | . | A | . | . | A | . | . | . | . | G | . | . | . | . | . | . | . | . | . | . | . | . | A | . | . | . | . | . | . | . | . | . | . | . | . | . | . |
| H52 | . | . | . | . | . | . | . | . | . | . | . | . | . | . | . | . | . | . | . | . | A | . | . | A | . | T | . | . | G | . | . | . | . | . | . | . | . | . | . | . | . | A | . | . | . | . | . | . | . | . | . | . | . | . | . | . |
| H53 | . | . | T | G | . | T | . | C | C | G | . | . | T | A | G | . | . | T | . | T | A | T | C | A | . | T | T | C | . | A | G | T | C | . | C | . | . | C | C | T | C | A | C | . | G | . | T | C | . | . | T | . | C | T | A | . |
| H54 | . | . | T | G | . | T | . | C | C | G | . | . | T | A | G | . | . | T | . | T | A | T | C | A | . | T | T | C | . | A | G | T | C | . | C | . | . | C | C | T | C | A | C | . | . | . | T | C | . | . | T | . | C | T | A | . |
| H55 | . | . | . | . | . | . | . | C | C | G | . | . | T | A | . | . | . | T | . | T | A | T | C | A | . | T | T | C | . | A | . | T | C | T | C | . | . | C | C | T | C | A | C | . | . | G | T | C | . | . | T | . | C | T | A | C |
| H56 | . | . | . | . | . | . | . | C | C | G | . | . | T | A | G | . | . | T | . | T | A | T | C | A | . | T | T | C | . | A | G | . | C | T | C | . | . | C | C | T | C | A | C | . | . | . | T | C | . | . | T | . | C | T | A | C |
| H57 | . | . | . | . | . | . | . | C | C | G | . | . | T | A | . | . | . | T | . | T | A | T | C | A | T | T | T | C | . | A | . | T | C | T | C | . | . | C | C | T | C | A | C | . | . | . | T | C | . | . | T | . | C | T | A | C |
| H58 | . | . | . | G | . | . | . | C | C | G | . | . | T | A | G | . | . | T | . | T | A | T | C | A | . | . | T | C | . | A | G | T | C | T | C | . | . | C | C | T | C | A | C | . | . | . | T | C | C | . | T | . | C | T | A | T |

|  |  |  |  |  |  |  |  |  |  |  |  |  |  |  |  |  |  |  |  |  |  |  |  |  |  |  |  |  |  |  |  |  |  |  |  |  |  |  |  |  |  |  |  |  | 1 | 1 | 1 | 1 | 1 | 1 | 1 | 1 | 1 | 1 | 1 | 1 |
| --- | --- | --- | --- | --- | --- | --- | --- | --- | --- | --- | --- | --- | --- | --- | --- | --- | --- | --- | --- | --- | --- | --- | --- | --- | --- | --- | --- | --- | --- | --- | --- | --- | --- | --- | --- | --- | --- | --- | --- | --- | --- | --- | --- | --- | --- | --- | --- | --- | --- | --- | --- | --- | --- | --- | --- | --- |
|  | 5 | 5 | 5 | 5 | 6 | 6 | 6 | 6 | 6 | 6 | 6 | 6 | 6 | 6 | 6 | 7 | 7 | 7 | 7 | 7 | 7 | 7 | 7 | 7 | 7 | 7 | 8 | 8 | 8 | 8 | 9 | 9 | 9 | 9 | 9 | 9 | 9 | 9 | 9 | 9 | 9 | 9 | 9 | 9 | 0 | 0 | 0 | 0 | 0 | 0 | 0 | 1 | 1 | 1 | 1 | 1 |
| H | 7 | 8 | 8 | 9 | 1 | 1 | 6 | 6 | 6 | 7 | 7 | 7 | 9 | 9 | 9 | 0 | 1 | 2 | 2 | 3 | 3 | 4 | 4 | 4 | 8 | 9 | 4 | 4 | 5 | 9 | 0 | 0 | 0 | 2 | 3 | 4 | 6 | 6 | 7 | 7 | 7 | 8 | 8 | 9 | 1 | 1 | 3 | 3 | 3 | 3 | 4 | 0 | 2 | 6 | 6 | 7 |
|  | 6 | 2 | 8 | 1 | 2 | 5 | 0 | 3 | 7 | 1 | 5 | 8 | 3 | 6 | 7 | 2 | 7 | 0 | 3 | 2 | 8 | 2 | 4 | 5 | 6 | 5 | 6 | 9 | 3 | 4 | 0 | 1 | 9 | 7 | 3 | 5 | 6 | 9 | 0 | 5 | 8 | 4 | 7 | 9 | 0 | 4 | 0 | 2 | 5 | 8 | 4 | 6 | 5 | 1 | 6 | 0 |
| H1 | A | C | T | T | T | T | A | T | C | C | T | A | A | T | T | T | T | T | A | A | A | A | T | T | C | A | G | A | G | A | A | C | T | T | A | C | A | A | T | T | T | T | A | C | C | T | G | C | A | C | A | A | C | G | T | C |
| H2 | . | . | . | . | . | . | . | . | . | . | . | . | . | . | . | . | . | . | . | . | . | . | . | C | . | . | A | . | . | . | . | . | . | . | . | . | . | . | . | . | . | . | . | . | . | . | . | . | . | . | . | . | . | . | . | . |
| H3 | . | . | . | . | . | . | T | . | . | . | . | . | . | . | . | . | . | . | . | . | . | . | . | . | . | . | . | . | . | . | G | . | . | . | . | . | . | . | . | . | . | . | . | . | T | . | . | . | . | . | . | . | . | . | . | . |
| H4 | . | . | . | . | . | . | G | . | . | . | . | . | . | . | . | . | . | . | . | . | . | . | . | . | . | . | . | . | . | . | . | . | . | . | . | . | . | . | . | . | . | . | . | . | . | . | . | . | . | . | . | . | . | . | . | . |
| H5 | . | . | . | . | . | . | . | . | . | . | . | . | . | . | . | . | . | . | . | . | . | . | . | . | . | . | . | . | . | . | . | . | . | . | . | . | . | . | . | . | . | . | . | . | . | C | . | . | . | . | . | . | . | . | . | . |
| H6 | . | . | . | . | . | . | . | . | . | . | . | . | . | . | . | . | . | . | . | . | . | . | . | . | . | . | . | . | . | . | . | . | . | . | . | . | . | . | . | . | . | . | . | . | . | . | . | . | . | . | . | . | . | . | . | . |
| H7 | . | . | . | . | . | . | . | . | . | . | . | . | . | . | . | . | . | . | . | . | . | . | . | . | . | . | . | . | . | . | . | T | . | . | . | . | . | . | . | . | . | T | A | . | . | . | . | . | . | . | . | . | . | G | T | C |
| H8 | . | . | . | . | . | C | . | . | . | . | . | . | . | . | . | . | . | . | . | . | . | . | . | . | . | . | . | . | . | . | . | . | . | . | . | C | . | . | . | . | . | . | . | . | . | . | . | . | . | . | . | . | . | . | . | T |
| H9 | . | . | . | . | . | . | . | . | . | . | . | . | . | . | . | . | . | . | . | . | . | . | . | . | . | . | A | . | . | . | . | . | . | . | . | . | . | . | . | . | . | . | . | . | . | . | . | . | . | . | . | . | . | . | . | . |
| H10 | . | . | . | . | . | . | . | . | . | . | . | . | . | . | . | . | . | . | . | . | . | . | . | . | . | T | . | . | . | . | . | . | . | . | . | . | . | . | . | . | T | . | . | . | . | . | . | . | . | . | . | . | . | G | T | C |
| H11 | . | . | . | . | . | . | . | . | . | . | . | G | . | . | . | . | . | . | . | . | . | . | . | . | . | . | . | . | . | . | . | . | . | . | . | . | . | . | . | . | T | . | . | . | . | . | . | . | . | . | . | . | . | . | . | T |
| H12 | . | . | . | . | . | . | . | . | . | . | . | . | . | . | . | . | . | . | . | . | . | . | . | . | . | . | A | . | . | . | . | . | . | . | . | . | . | . | . | . | . | . | . | C | . | . | . | . | . | . | . | . | . | A | . | . |
| H13 | . | . | . | . | . | . | . | . | . | . | . | . | . | . | . | . | . | . | G | . | . | . | . | . | . | . | . | . | . | . | . | . | . | . | . | . | . | . | . | . | . | . | . | . | . | . | . | . | . | . | . | . | . | . | . | . |
| H14 | . | . | . | . | . | . | . | . | . | . | . | . | . | . | . | . | . | . | . | . | . | . | . | . | . | . | . | . | . | . | . | . | . | . | . | . | . | . | . | . | . | . | . | . | . | . | . | . | . | . | . | . | . | . | . | . |
| H15 | . | . | . | . | . | . | . | . | . | . | . | . | . | . | . | . | . | . | . | . | . | . | . | . | . | . | . | . | . | . | . | . | . | . | . | . | . | . | . | . | . | . | . | . | . | . | . | . | . | . | . | . | . | . | . | . |
| H16 | . | . | . | . | . | . | . | . | . | . | . | . | . | . | . | . | . | . | . | . | . | . | . | . | . | . | . | . | . | . | . | . | . | . | . | . | . | . | . | . | . | . | . | . | . | . | . | . | . | . | . | . | . | . | . | . |
| H17 | . | . | . | . | . | . | . | . | . | . | . | . | . | . | . | . | . | . | . | . | . | . | . | . | . | . | . | . | . | . | . | . | . | . | . | . | . | . | . | . | . | . | . | . | . | . | . | . | . | . | . | . | . | . | . | . |
| H18 | . | . | . | . | . | . | . | . | . | . | . | . | . | . | . | . | . | . | . | . | . | . | . | . | . | . | . | . | . | . | . | . | . | . | . | . | . | . | . | . | . | . | . | . | . | . | . | . | . | . | . | . | . | . | . | . |
| H19 | . | . | . | . | . | . | . | . | . | . | . | . | . | . | . | . | . | . | . | . | . | . | . | . | . | . | . | . | . | . | . | . | . | . | . | . | . | . | . | . | . | . | . | . | . | . | . | . | . | . | . | . | . | . | . | . |
| H20 | . | . | . | . | . | . | . | . | . | . | . | . | . | . | . | . | . | . | . | . | . | . | . | . | . | . | . | . | . | . | . | . | . | . | . | . | . | . | . | . | . | . | . | . | . | . | . | . | . | . | . | . | . | A | . | . |
| H21 | . | . | . | . | . | . | . | . | . | . | . | . | . | . | . | . | . | . | . | . | . | . | . | . | . | . | . | . | . | . | . | . | . | . | . | . | . | . | . | . | . | . | . | . | . | . | . | . | . | . | . | . | . | . | . | . |
| H22 | . | . | . | . | . | . | . | . | . | . | . | . | . | . | . | . | . | . | . | . | . | . | . | . | . | . | A | . | . | . | . | . | . | . | . | . | . | . | . | . | . | . | . | . | . | . | . | . | . | . | . | . | . | . | . | . |
| H23 | . | . | . | . | . | . | . | . | T | . | . | . | . | . | . | . | . | . | . | . | . | . | . | . | . | . | . | . | . | . | . | . | . | . | . | . | . | . | . | . | . | . | . | . | . | . | A | . | . | . | . | . | . | . | . | . |
| H24 | . | . | . | . | . | . | . | . | . | . | . | . | . | . | . | . | . | . | . | . | . | . | . | . | . | . | . | . | . | . | . | . | . | . | . | . | G | . | . | . | . | . | . | . | . | . | . | . | . | . | . | . | . | . | . | . |
| H25 | . | C | . | . | . | . | . | . | . | . | . | . | . | . | . | . | . | . | . | . | . | . | . | . | . | . | . | . | . | . | . | . | . | . | . | . | . | . | . | . | . | . | . | . | . | . | . | . | . | . | . | . | . | . | . | . |
| H26 | . | . | T | . | . | T | . | . | T | . | . | . | . | T | . | T | . | . | . | . | . | . | . | . | . | . | . | . | . | . | . | . | . | . | . | . | . | . | . | . | . | . | . | . | . | . | . | . | . | . | . | . | . | . | . | C |
| H27 | . | . | . | . | . | . | . | . | . | . | . | . | . | . | . | . | . | . | . | . | . | . | . | . | . | . | . | . | . | . | . | . | . | . | . | . | . | . | . | . | . | . | . | . | . | . | . | . | . | . | . | . | . | . | . | . |
| H28 | . | . | . | . | . | . | . | . | . | . | . | . | . | . | T | . | . | . | . | . | . | . | . | . | . | . | . | . | . | . | . | . | . | . | . | . | . | . | . | . | . | . | . | . | . | . | . | . | . | . | G | . | . | . | . | . |
| H29 | . | . | . | . | . | . | G | . | . | . | . | . | . | . | . | . | . | . | . | G | . | . | . | . | . | . | . | . | . | . | . | . | . | C | G | . | . | . | . | . | T | . | . | . | . | . | . | . | G | . | . | . | . | . | . | T |
| H30 | . | . | . | . | . | . | G | . | . | . | . | . | . | . | . | . | . | . | . | G | . | . | . | . | . | . | . | . | . | . | . | . | . | . | G | . | . | . | . | . | . | . | . | . | . | . | . | . | G | . | . | . | . | . | . | T |
| H31 | . | . | . | . | . | . | G | . | . | . | . | . | . | . | . | . | . | . | . | G | . | . | . | . | . | . | . | . | . | . | . | . | . | . | G | . | . | . | . | . | T | . | . | . | . | . | . | . | G | . | . | . | . | . | . | T |
| H32 | . | . | . | . | . | . | . | . | . | T | . | . | . | . | . | . | . | . | . | G | . | . | . | . | . | . | . | . | . | . | . | . | . | . | G | . | . | . | T | . | . | . | . | . | . | . | . | . | G | . | . | . | . | . | C | T |
| H33 | . | . | . | . | . | . | . | . | . | . | . | . | . | . | . | . | . | . | . | G | . | . | . | . | . | . | . | . | . | . | . | . | . | . | G | . | . | . | . | . | . | . | . | . | . | . | . | . | G | . | . | . | . | . | . | T |
| H34 | . | . | . | . | . | . | . | . | . | . | . | . | . | . | . | . | . | . | . | G | . | . | . | . | . | . | . | . | . | . | . | . | . | . | G | . | . | . | . | . | . | . | . | . | . | . | . | . | G | . | . | . | . | . | . | T |
| H35 | . | . | . | . | . | . | . | . | . | . | . | . | . | . | . | . | . | . | . | G | . | . | . | . | . | . | . | . | . | . | . | . | . | . | G | . | . | . | . | . | . | . | . | . | . | . | . | . | G | . | . | . | . | . | . | T |
| H36 | . | . | . | . | . | . | G | . | . | . | . | . | . | . | . | . | . | . | . | G | . | . | . | . | . | . | . | . | . | . | . | . | . | C | G | . | . | . | . | . | . | . | . | . | . | . | . | . | G | . | . | . | . | . | . | T |
| H37 | . | . | . | . | . | . | . | . | . | . | . | . | . | C | . | . | . | . | . | G | . | . | . | . | . | . | G | . | . | . | . | . | . | . | G | . | . | . | . | . | . | . | . | . | . | . | . | . | G | . | . | . | . | . | . | T |
| H38 | . | . | . | . | . | . | . | . | . | . | . | . | . | . | T | . | . | . | . | G | . | . | . | . | . | . | . | . | . | . | . | . | . | . | G | . | . | . | . | . | . | . | . | . | . | . | . | . | G | . | . | . | . | . | . | T |
| H39 | . | . | . | . | . | . | G | . | . | . | . | . | . | . | . | . | . | . | . | G | . | . | . | . | . | . | . | . | . | . | . | . | . | . | G | . | . | . | . | . | . | . | . | . | . | . | . | . | G | . | . | . | . | . | . | T |
| H40 | . | . | . | . | . | . | G | . | . | . | . | . | . | . | . | . | . | . | . | . | . | . | . | . | . | . | A | . | . | . | . | . | . | . | G | . | . | . | . | . | . | . | . | . | . | . | . | . | G | . | . | . | . | . | . | T |
| H41 | . | . | . | . | . | . | G | . | . | . | . | . | . | . | . | . | . | . | . | G | . | . | . | . | . | . | . | . | . | . | . | . | . | . | G | . | . | . | . | . | . | . | . | . | . | . | . | . | G | . | . | . | . | . | . | T |
| H42 | . | . | . | . | . | . | G | . | . | . | . | . | . | . | . | . | . | . | . | G | . | . | . | . | . | . | . | . | . | . | . | . | . | . | G | . | . | . | . | . | . | . | . | . | . | . | . | . | G | . | G | . | . | A | . | T |
| H43 | . | . | . | . | . | . | G | . | . | . | . | . | . | . | . | . | . | . | . | G | . | . | . | . | . | . | . | . | . | . | . | . | . | . | G | . | . | . | . | . | . | . | . | . | . | . | . | . | G | . | . | . | T | . | . | T |
| H44 | . | . | . | . | . | . | G | . | . | . | . | . | . | . | . | . | . | . | . | G | . | . | . | . | . | . | . | . | . | . | . | . | . | . | G | . | . | . | . | . | . | . | . | . | . | . | . | . | . | . | . | . | . | . | . | T |
| H45 | . | . | . | . | . | . | . | . | . | . | . | . | . | . | . | . | . | . | . | G | . | . | . | . | . | . | . | . | . | . | . | . | . | . | G | . | . | . | . | . | . | . | . | . | . | . | . | . | G | . | . | . | . | . | . | T |
| H46 | . | . | . | . | . | . | G | . | . | . | . | . | . | . | . | . | . | . | . | G | . | . | . | . | . | . | . | . | A | . | . | . | . | . | G | . | . | . | . | . | . | . | . | . | . | . | . | . | G | . | . | . | . | . | . | T |
| H47 | . | . | . | . | . | . | . | . | . | . | . | . | . | . | . | . | . | . | . | G | . | . | . | . | . | . | . | . | . | . | . | . | . | . | G | . | . | . | . | . | . | . | . | . | . | . | . | . | G | . | . | . | . | . | . | T |
| H48 | . | . | . | . | . | . | . | . | . | . | . | . | . | . | . | . | . | . | . | G | . | . | . | . | . | . | . | . | . | . | . | . | . | . | G | . | . | . | . | . | . | . | . | . | . | . | A | . | G | . | . | . | . | . | . | T |
| H49 | . | . | . | . | . | . | G | . | . | . | . | . | G | . | . | . | . | . | . | G | . | . | . | . | . | . | . | . | . | . | . | . | . | . | G | . | . | . | . | . | . | . | . | . | . | . | . | . | G | . | . | . | . | . | . | T |
| H50 | . | . | . | . | . | . | G | . | . | . | . | . | . | . | . | . | . | . | . | G | . | . | . | . | . | . | . | . | . | . | . | . | . | . | G | . | . | . | . | . | . | . | . | . | . | . | . | . | G | . | . | . | . | . | . | T |
| H51 | . | . | . | . | . | . | G | . | . | . | . | . | . | . | . | C | . | . | . | G | . | . | . | . | . | . | A | . | . | . | . | . | . | . | G | . | . | . | . | . | . | . | . | . | . | . | . | . | G | . | . | . | . | . | . | T |
| H52 | . | . | . | . | . | . | G | . | . | . | . | . | . | . | . | . | . | . | . | G | . | . | . | . | . | . | . | . | . | . | . | . | . | . | G | . | . | . | . | . | . | . | . | . | . | . | . | . | G | . | . | . | . | . | . | T |
| H53 | T | T | C | C | . | A | . | C | T | . | C | . | . | . | C | G | C | . | . | G | G | G | C | . | T | . | A | G | . | T | . | . | . | . | . | T | . | G | C | G | A | C | . | T | . | C | . | A | . | A | . | C | . | A | . | T |
| H54 | T | T | C | C | . | A | . | C | T | . | C | . | . | . | C | G | C | . | . | G | G | G | C | . | T | . | A | . | . | T | . | . | . | . | . | T | . | G | C | G | A | C | . | T | . | C | . | A | . | A | . | C | . | A | . | T |
| H55 | T | T | C | C | . | A | . | C | T | . | C | . | . | . | C | G | C | . | . | . | G | G | C | . | T | . | A | . | . | T | . | . | . | . | . | T | . | G | C | G | G | C | G | T | . | C | . | G | . | A | . | C | . | A | . | T |
| H56 | T | T | C | C | . | A | . | C | T | . | C | . | . | . | C | G | C | . | . | G | G | G | C | . | T | . | A | . | . | T | . | . | . | . | . | T | . | G | C | A | A | C | G | T | . | C | . | A | . | A | . | C | . | A | . | T |
| H57 | T | T | C | C | C | A | . | C | T | . | C | . | . | . | C | A | C | . | . | G | G | G | C | . | T | . | A | . | . | T | . | . | . | . | . | T | . | G | C | G | A | . | G | T | . | C | . | A | . | A | . | C | . | A | . | T |
| H58 | T | T | C | C | . | A | . | C | T | . | C | . | . | . | C | A | C | C | . | G | G | G | C | . | T | . | A | . | . | T | . | . | C | . | . | T | . | G | C | A | A | T | G | T | C | C | G | A | A | A | A | C | . | A | . | T |
